# Supplementary material for: The Effect of Stimulants on Nectar Composition, Flowering, and Seed Yield of Common Buckwheat (Fagopyrum esculentum Moench)
Source: Int J Mol Sci. 2023 Aug 16;24(16):12852. doi: 10.3390/ijms241612852 (PMC10454428; doi:10.3390/ijms241612852)
Supplement: Supplementary file 1 [file ijms-24-12852-s001.zip › Suppl. Table 1.pdf]

Supplementary Table S1. Effect of studied stimulants applied at the beginning of flowering (I term) or at full flowering stage (II term) on volume of nectar in flowers of buckwheat PA15 plants [µl/50 flowers], nectar weight [mg/50 flowers], glycerol content, total sugars [mg cm^{-3}] and the percentage share of individual sugars in the total pool of soluble carbohydrates [%]. Means (n=3) for each parameter within each term marked with the same letter do not differ significantly (multiple Duncan's test; p<0.05).

Stimulant	Volume of nectar [µl/50 flowers]	Mass of nectar [mg/50 flowers]	Glycerol [mg cm^{-3}]	Percentage content of individual sugars [%]				Total sum of sugars [mg cm^{-3}]
				Glucose	Fructose	Sucrose	Fructans	
**I term**								
Control	66.0 b	68.39 a	0.86 b	13.17 a	10.77 a	1.24 b	4.55 ab	30.67 a
NAA	61.2 c	63.80 a	1.21 ab	5.37 c	6.74 c	0.79 c	2.86 c	16.57 c
BAP	69.1 b	70.31 a	0.90 ab	7.91 b	8.93 b	1.69 b	3.71 b	23.04 b
NaCl	76.7 a	66.50 a	1.00 ab	7.58 b	6.26 c	1.16 bc	4.18 b	20.06 b
ASAHI SL	61.1 c	62.16 a	0.92 b	12.79 a	10.34 a	2.86 a	5.01 a	32.01 a
Cysteine	60.9 c	62.77 a	0.95 b	12.74 a	11.91 a	3.72 a	3.66 b	33.04 a
TYTANIT	61.2 c	62.88 a	1.22 ab	10.81 a	10.65 a	2.16 b	3.70 b	28.22 ab
GA_3	52.3 d	53.62 b	1.10 ab	10.31 a	8.76 b	1.69 b	4.50 ab	26.23 ab
Putrescine	60.5 c	62.68 a	1.40 a	10.27 a	10.92 a	1.65 b	3.65 b	27.52 ab
**II term**								
Control	63.9 a	65.25 b	1.06 ab	4.93 d	6.32 c	0.55 d	2.59 ab	15.26 c
NAA	51.7 b	53.25 c	1.08 ab	4.69 d	5.80 cd	0.41d	2.01 b	13.74 c
BAP	61.2 a	63.01 b	1.12 ab	5.61 c	7.31 b	0.99 c	1.68 c	16.34 bc
NaCl	65.4 a	79.07 a	0.89 b	4.87 d	6.29 c	0.62 d	1.72 c	14.37 cd
ASAHI SL	59.6 a	61.52 b	1.19 ab	5.39 c	6.92 c	0.37 d	2.59 ab	16.18 bc
Cysteine	53.8 b	54.73 c	0.88 b	6.09 b	6.69 c	2.02 b	2.26 b	17.96 b
TYTANIT	65.9 a	67.28 a	1.04 ab	2.13 e	2.84 e	0.21 e	2.01 bc	8.08 e
GA_3	28.7 c	29.46 d	1.41 a	7.71 a	9.29 a	3.08 a	3.03 a	24.03 a
Putrescine	53.2 b	54.46 c	0.90 b	4.21 d	5.41 d	0.40 e	2.86 a	13.75 cd

BAP – 6-benzylaminopurine; NAA – 1-naphthaleneacetic acid; cysteine, GA3 – gibberellic acid, NaCl – sodium chloride), ASAHI SL and TYTANIT – commercial preparates. Data marked with green colour show positive effect of stimulant comparing to control value separative for each control.
