# Supplementary material for: The Effect of Stimulants on Nectar Composition, Flowering, and Seed Yield of Common Buckwheat (Fagopyrum esculentum Moench)
Source: Int J Mol Sci. 2023 Aug 16;24(16):12852. doi: 10.3390/ijms241612852 (PMC10454428; doi:10.3390/ijms241612852)
Supplement: Supplementary file 1 [file ijms-24-12852-s001.zip › Suppl. Table 2.pdf]

Supplementary Table S2. Effect of studied stimulants applied at the beginning of flowering (I term) or at full flowering stage (II term) on volume of nectar in flowers of buckwheat PA16 plants [µl/50 flowers], nectar weight [mg/50 flowers], glycerol content, total sugars [mg cm$^{-3}$] and the percentage share of individual sugars in the total pool of soluble carbohydrates [%]. Means (n=3) for each parameter within each term marked with the same letter do not differ significantly (multiple Duncan's test; $p<0.05$).

| Stimulant | Volume of nectar [µl/50 flowers] | Mass of nectar [mg/50 flowers] | Glycerol [g cm$^{-3}$] | Percentage content of individual sugars [%] | | | | Total sum of sugars [mg cm$^{-3}$] |
|---|---|---|---|---|---|---|---|---|
| | | | | Glucose | Fructose | Sucrose | Fructans | |
| **I term** | | | | | | | | |
| Control | 63.1 a | 65.1 a | 1.23 b | 6.80 a | 8.61a | 0.70 b | 3.11 a | 20.06 a |
| NAA | 63.6 a | 65.3 a | 1.27 b | 4.76 c | 6.39 b | 0.30 e | 3.54 a | 15.81 d |
| BAP | 40.2 c | 41.5 d | 1.20 b | 5.11bc | 7.12 ab | 0.16 f | 3.04 a | 16.24 cd |
| NaCl | 50.5 b | 52.0 b | 1.22 b | 5.31 b | 6.91 ab | 0.59 c | 3.44 a | 17.05 c |
| ASAHI SL | 53.9 b | 55.2 bc | 1.27 b | 6.04 a | 7.89 a | 0.86 a | 2.67 b | 18.25 bc |
| Cysteine | 47.4 bc | 47.9 cd | 1.29 b | 7.25 a | 8.91 a | 0.52 c | 3.73 a | 21.08 a |
| TYTANIT | 57.5 ab | 59.5 a | 1.42 a | 5.73 bc | 7.48 a | 0.51 c | 3.00 a | 17.48 c |
| $GA_3$ | 50.9 b | 52.1 b | 1.48 a | 5.89 bc | 8.04 a | 0.41d | 3.21 a | 18.40 bc |
| Putrescine | 67.1 a | 69.3 a | 1.36 ab | 4.56 c | 6.16 b | 0.13 f | 3.74 a | 15.37 d |
| **II term** | | | | | | | | |
| Control | 54.3 ab | 55.9 ab | 1.25 b | 5.95 b | 7.60 a | 1.95 b | 2.13 a | 18.44 b |
| NAA | 62.4 a | 64.3 a | 1.22 b | 6.48 a | 8.29 a | 1.53 b | 2.84 a | 19.92 a |
| BAP | 51.7 ab | 51.9 bc | 1.23 b | 6.32 ab | 7.26 a | 1.50 b | 2.11 a | 18.02 b |
| NaCl | 45.3 b | 46.5 c | 1.16 bc | 5.21 b | 6.62 b | 1.55 b | 2.27 a | 16.55 cd |
| ASAHI SL | 44.9 b | 46.0 c | 1.46 a | 6.45 a | 7.86 a | 1.14 c | 2.90 a | 19.28 ab |
| Cysteine | 59.6 a | 60.7 a | 1.22 b | 6.19 ab | 7.51 a | 2.70 a | 2.59 a | 19.86 ab |
| TYTANIT | 51.7 ab | 52.6 b | 1.09 c | 6.56 a | 8.03 a | 1.30 b | 1.75 b | 18.53 b |
| $GA_3$ | 54.0 ab | 54.7 ab | 1.21 b | 7.07 a | 8.03 a | 2.40 a | 2.39 a | 20.82 a |
| Putrescine | 62.5 a | 63.2 a | 1.10 bc | 4.07 c | 5.41 c | 0.80 d | 2.44 a | 13.61 e |

BAP – 6-benzylaminopurine; NAA – 1-naphthaleneacetic acid; cysteine, GA3 – gibberellic acid, NaCl – sodium chloride), ASAHI SL and TYTANIT – commercial preparates. Data marked with green colour show positive effect of stimulant comparing to control value separative for each control.
