# Supplementary material for: The Effect of Stimulants on Nectar Composition, Flowering, and Seed Yield of Common Buckwheat (Fagopyrum esculentum Moench)
Source: Int J Mol Sci. 2023 Aug 16;24(16):12852. doi: 10.3390/ijms241612852 (PMC10454428; doi:10.3390/ijms241612852)
Supplement: Supplementary file 1 [file ijms-24-12852-s001.zip › Suppl. Table 3.pdf]

Supplementary Table S3. Content of individual amino acids [nM cm^{-3}] and their percentage participation in total amino acid amount in the nectar of control plants of common buckwheat PA15 line collected at the beginning of flowering (I term) or at full flowering stage (II term). Nectar was analysed in the open flowers able to fertilization. Means (n = 3) ± SE.

Amino-acid	I term	% of total content	II term	% of total content
Glycine	230 ± 20	0.67	352 ± 87	1.45
Alanine	1544 ±197	4.53	1451 ± 153	5.96
Valine	636 ± 52	1.87	687 ± 24	2.82
Leucine	1098 ± 69	3.22	1333 ± 69	5.48
Isleucine	859 ± 45	2.52	837 ± 58	3.44
Phenylalanine	162 ± 24	0.48	326 ± 50	1.34
Tryptophan	225 ± 16	0.66	472 ± 46	1.94
Tyrosine	836 ± 12	2.45	875 ± 87	3.59
Proline	351 ± 35	1.03	81 ± 11	0.33
Histidine	485 ± 29	1.42	1832 ± 94	7.53
Serine	2998 ± 141	8.80	2422 ± 143	9.95
Threonine	1642 ± 170	4.82	1369 ± 138	5.62
Methionine	20.4 ± 4.8	0.06	28.3 ± 6.5	0.12
Arginine	369 ± 88	1.08	385 ± 50	1.58
Aspartic acid	1368 ± 147	4.01	1207 ± 155	4.96
Glutamine	17462 ± 145	51.25	7669 ± 103	31.50
Glutamic acid	2484 ± 143	7.29	1719 ± 166	7.06
Asparagine	695 ± 31	2.04	608 ± 39	2.50
Lysine	610 ± 31	1.79	689 ± 58	2.83
Total	34074,4	100	24342,3	100
