# Supplementary material for: The Effect of Stimulants on Nectar Composition, Flowering, and Seed Yield of Common Buckwheat (Fagopyrum esculentum Moench)
Source: Int J Mol Sci. 2023 Aug 16;24(16):12852. doi: 10.3390/ijms241612852 (PMC10454428; doi:10.3390/ijms241612852)
Supplement: Supplementary file 1 [file ijms-24-12852-s001.zip › Suppl. Table 4.pdf]

Supplementary Table S4. Content of individual amino acids [nM cm^{-3}] and their percentage participation in total amino acid amount in the nectar of control plants of common buckwheat PA16 line collected at the beginning of flowering (I term) or at full flowering stage (II term). Nectar was analysed in the open flowers able to fertilization. Means (n = 3) ± SE.

Amino acid	I term	% of total content	II term	% of total content
Glycine	804 ± 58	1.54	230 ± 46	1.04
Alanine	1812 ± 155	3.47	958 ± 21	4.31
Valine	851 ± 96	1.63	663 ± 48	2.99
Leucine	1448 ± 35	2.77	1567 ± 103	7.06
Isleucine	1112 ± 55	2.13	1076 ± 69	4.84
Phenylalanine	457 ± 77	0.87	705 ± 96	3.17
Tryptophan	588 ± 64	1.13	445 ± 66	2.00
Tyrosine	1174 ± 59	2.25	867 ± 12	3.90
Proline	120 ± 12	0.23	57 ± 4	0.26
Histidine	717 ± 34	1.37	1052 ± 114	4.74
Serine	4192 ± 195	8.03	2152 ± 99	9.69
Threonine	3213 ± 44	6.15	1374 ± 48	6.19
Methionine	95 ± 14	0.18	30 ± 11	0.14
Asparagine	850 ± 10	1.63	606 ± 16	2.73
Aspartic acid	1712 ± 32	3.28	1337 ± 48	6.02
Glutamine	28580 ± 639	54.72	6653 ± 32	29.95
Glutamic acid	2487 ± 110	4.76	1643 ± 23	7.40
Arginine	899 ± 38	1.72	281 ± 35	1.27
Lysine	1120 ± 48	2.14	514 ± 64	2.31
Total	52231	100	22210	100
