# Supplementary material for: The Effect of Stimulants on Nectar Composition, Flowering, and Seed Yield of Common Buckwheat (Fagopyrum esculentum Moench)
Source: Int J Mol Sci. 2023 Aug 16;24(16):12852. doi: 10.3390/ijms241612852 (PMC10454428; doi:10.3390/ijms241612852)
Supplement: Supplementary file 1 [file ijms-24-12852-s001.zip › Suppl. Table 5A.pdf]

Supplementary Table S5A. Effect of studied stimulants applied at the beginning of flowering (I term) or at full flowering stage (II term) on content [nM cm^{-3}] following amino-acids: glycine, alanine, valine, leucine, isoleucine, phenylalanine, tryptophan, tyrosine, proline and histidine in the nectar of common buckwheat line PA15. Nectar was analysed in the open flowers able to fertilization. Means (n = 3) ± SE.

Treatment	Glycine	Alanine	Valine	Leucine	Isleucine	Phenylalanine	Tryptophan	Tyrosine	Proline	Histidine
I term										
Control	230 ± 20	1544 ± 197	636 ± 52	1098 ± 69	859 ± 45	162 ± 24	225 ± 16	836 ± 12	351 ± 35	485 ± 29
BAP	234 ± 56	1136 ± 126	687 ± 95	1394 ± 119	1037 ± 120	254 ± 50	248 ± 31	774 ± 89	97 ± 22	548 ± 38
NAA	277 ± 49	1426 ± 132	1047 ± 105	1624 ± 144	1245 ± 181	507 ± 31	612 ± 42	912 ± 86	98 ± 21	779 ± 86
GA_3	146 ± 16	1557 ± 80	473 ± 95	1130 ± 171	871 ± 87	248 ± 51	285 ± 58	791 ± 82	69 ±10	360 ± 48
Cysteine	241 ± 34	1167 ± 139	970 ± 87	1866 ± 178	1480 ±109	262 ± 50	518 ± 98	1158 ± 123	108 ± 10	834 ± 99
Putrescine	278 ± 78	1484 ± 160	841 ± 80	1459 ± 164	1113 ± 116	176 ± 68	400 ±135	1177 ± 123	104 ± 34	858 ± 66
NaCl	163 ± 21	1226 ±115	552 ± 83	1220 ± 162	808 ± 99	245 ± 31	201 ± 66	706 ± 98	171 ± 29	458 ± 41
ASAHI	134 ± 23	1001 ±149	617 ± 51	1014 ± 105	813 ± 102	177 ± 18	254 ± 64	544 ± 47	131 ± 17	449 ± 56
TYTANIT	143 ± 11	909 ±195	654 ± 44	1573 ± 181	1203 ± 113	184 ± 52	279 ± 86	663 ± 69	89 ± 22	678 ± 46
II term										
Control	352 ±87	1451 ± 153	687 ± 24	1333 ± 69	837 ± 58	326 ± 50	472 ± 46	875 ± 87	81 ± 11	1832 ± 94
BAP	483 ± 20	1429 ±10	689 ±95	1498 ±127	992 ± 27	334 ± 47	509 ± 74	709 ± 95	74 ± 3	1741 ± 44
NAA	364 ± 72	950 ± 97	710 ± 63	1517 ± 125	916 ± 115	505 ± 51	580 ± 50	972 ± 70	61 ± 6	779 ± 86
GA_3	254 ± 53	1256 ± 102	485 ± 42	1267 ± 98	754 ± 52	294 ± 41	371 ± 98	684 ± 63	72 ± 6	693 ± 99
Cysteina	343 ± 90	964 ± 15	699 ± 46	1431 ± 131	995 ± 93	310 ± 47	614 ± 77	1025 ± 34	38 ± 4	1841 ± 85
Putrescine	319 ± 93	1029 ±108	561 ± 83	923 ± 140	610 ± 148	296 ± 73	446 ± 35	661 ± 32	109 ± 10	1010 ± 69
NaCl	421 ±84	1204 ± 71	846 ± 47	1389 ± 110	971 ± 141	380 ± 42	423 ± 22	865 ± 66	58 ± 12	1798 ± 90
ASAHI	348 ± 31	1204 ± 65	592 ± 67	992 ± 129	635 ± 73	207 ± 39	419 ± 23	656 ± 89	85 ± 23	1904 ± 143
TYTANIT	124 ± 36	980 ± 83	409 ± 87	890 ± 111	1203 ± 113	404 ± 58	365 ± 86	584 ± 94	67 ± 9	704 ± 77

BAP – 6-benzylaminopurine; NAA – 1-naphthaleneacetic acid; cysteine, GA_3 – gibberellic acid, NaCl – sodium chloride), ASAHI SL and TYTANIT – commercial preparates. Data marked with green colour show positive effect of stimulant comparing to control value separative for each control.
