# Supplementary material for: The Effect of Stimulants on Nectar Composition, Flowering, and Seed Yield of Common Buckwheat (Fagopyrum esculentum Moench)
Source: Int J Mol Sci. 2023 Aug 16;24(16):12852. doi: 10.3390/ijms241612852 (PMC10454428; doi:10.3390/ijms241612852)
Supplement: Supplementary file 1 [file ijms-24-12852-s001.zip › suppl. Table 5B.pdf]

Supplementary Table S5B. Effect of studied stimulants applied at the beginning of flowering (I term) or at full flowering stage (II term) on content [nM cm^{-3}] following amino-acids: serine, threonine, methionine, arginine, aspartic acid, glutamine, glutamic acid, asparagine, lysine in the nectar of common buckwheat line PA16. Nectar was analysed in the open flowers able to fertilization. Means (n = 3) ± SE.

Treatment	Serine	Threonine	Methionine	Arginine	Aspartic acid	Glutamine	Glutamic acid	Asparagine	Lysine
**I term**									
Control	2998 ± 141	1642 ± 170	20.4 ± 4.8	369 ± 88	1368 ± 147	17462 ± 145	2484 ± 43	695 ± 31	610 ± 40
BAP	3379 ± 139	1526 ± 189	15.7 ± 8.4	674 ± 103	2202 ± 189	23834 ± 343	2864 ± 41	728 ± 98	850 ± 59
NAA	5301 ± 158	3535 ± 337	96 ± 3.80	1006 ± 168	3050 ± 129	38939 ± 653	4020 ± 78	1260 ± 102	1192 ± 71
GA_3	2857 ± 10	1181 ± 225	8.3 ± 1.6	710 ± 56	1589 ± 130	13531 ± 930	2783 ± 58	494 ± 48	849 ± 24
Cysteine	3886 ± 347	2319 ± 158	20.3 ± 8.9	874 ± 54	1875 ± 143	17850 ± 122	3010 ± 110	835 ± 68	944 ± 28
Putrescine	2976 ± 146	1811 ± 116	19.6 ± 9.1	768 ± 58	1248 ± 168	14358 ± 188	2346 ± 143	679 ± 26	955 ± 73
NaCl	2858 ± 225	1055 ± 194	58 ± 15	431 ± 41	1310 ± 165	14567 ± 231	2267 ± 137	557 ± 77	515 ± 52
ASAHI	2900 ± 235	1246 ± 75	11.7 ± 5.9	567 ± 71	1864 ± 101	18610 ± 464	2864 ± 153	688 ± 34	617 ± 59
TYTANIT	2832 ± 280	1502 ± 153	6.8 ± 0.3	422 ± 45	1924 ± 117	14304 ± 787	2550 ± 150	484 ± 94	528 ± 32
**II term**									
Control	2422 ± 143	1369 ± 132	28.3 ± 6.5	385 ± 50	1207 ± 155	7669 ± 603	1719 ± 166	608 ± 39	689 ± 58
BAP	2581 ± 221	1101 ± 128	34.9 ± 8.4	327 ± 56	1440 ± 146	7406 ± 603	1991 ± 137	594 ± 39	764 ± 66
NAA	2674 ± 157	1255 ± 100	43.6 ± 8.8	344 ± 78	1692 ± 134	8041 ± 926	1868 ± 113	750 ± 45	520 ± 67
GA_3	2962 ± 148	1025 ± 98	19.3 ± 1.8	598 ± 99	1476 ± 116	9786 ± 789	2063 ± 197	524 ± 51	795 ± 47
Cysteina	2633 ± 133	1276 ± 10	61.5 ± 10.4	427 ± 91	1375 ± 43	9894 ± 491	2026 ± 153	681 ± 51	630 ± 37
Putrescine	1987 ± 107	1066 ± 109	22.4 ± 2.6	309 ± 40	1319 ± 204	6292 ± 211	1650 ± 105	536 ± 30	576 ± 24
NaCl	3234 ± 293	1925 ± 93	48.5 ± 8.9	465 ± 128	1676 ± 166	13217 ± 802	2113 ± 141	1124 ± 71	597 ± 85
ASAHI	2267 ± 245	1049 ±146	10.4 ± 2.7	319 ± 51	1314 ± 132	6025 ± 695	1738 ± 117	1432 ± 101	571 ± 62
TYTANIT	1376 ± 130	668 ± 167	17.3 ± 1.9	206 ± 85	884 ± 90	14304 ± 787	924 ± 61	458 ± 48	330 ± 92

BAP – 6-benzylaminopurine; NAA – 1-naphthaleneacetic acid; GA_3 – gibberellic acid, NaCl – sodium chloride), ASAHI SL and TYTANIT – commercial preparates. Data marked with green colour show positive effect of stimulant comparing to control value separative for each control.
