# Supplementary material for: The Effect of Stimulants on Nectar Composition, Flowering, and Seed Yield of Common Buckwheat (Fagopyrum esculentum Moench)
Source: Int J Mol Sci. 2023 Aug 16;24(16):12852. doi: 10.3390/ijms241612852 (PMC10454428; doi:10.3390/ijms241612852)
Supplement: Supplementary file 1 [file ijms-24-12852-s001.zip › Suppl. Table 6 A.pdf]

Supplementary Table S6A. Effect of studied stimulant applied at the beginning of flowering (I term) or at full flowering stage (II term) on content [nM cm^{-3}] following amino-acids: glycine, alanine, valine, leucine, isoleucine, phenylalanine, tryptophan, tyrosine, proline and histidine in the nectar of common buckwheat line PA16. Nectar was analysed in the open flowers able to fertilization. Means (n = 3) ± SE.

Treatment	Glycyne	Alanine	Valine	Leucine	Isleucine	Phenylalanine	Tryptophan	Tyrosine	Proline	Histidine
I term										
Control	804 ± 58	1812 ± 155	851 ± 96	1448 ± 35	1112 ± 55	457 ± 77	588 ± 64	1174 ± 59	120 ± 12	717 ± 34
BAP	307 ± 59	702 ± 53	291 ± 92	459 ± 93	346 ± 46	159 ± 56	124 ± 65	324 ± 35	35 ± 5	240 ± 96
NAA	480 ± 50	1195 ± 60	746 ± 91	1140 ± 133	935 ± 89	331 ± 38	358 ± 95	805 ± 40	118 ± 16	861 ± 61
GA_3	6093 ± 51	1194 ± 253	610 ± 91	908 ± 42	708 ± 82	425 ± 64	307 ± 39	712 ± 53	107 ± 14	503 ± 78
Cysteine	448 ± 47	1197 ± 107	622 ± 58	1146 ± 194	807 ± 99	330 ± 39	258 ± 58	829 ± 85	90 ± 10	608 ± 23
Putrescine	949 ± 61	1522 ± 13	1056 ± 99	1594 ± 165	1223 ± 160	533 ± 63	515 ± 86	943 ± 17	152 ± 8	958 ± 84
NaCl	550 ± 57	1276 ± 118	606 ± 86	1106 ± 195	844 ± 67	500 ± 66	401 ± 49	764 ± 18	63 ± 3	626 ± 86
ASAHI	560 ± 85	1236 ± 196	818 ± 71	1316 ± 175	901 ± 92	424 ± 68	423 ± 59	704 ± 92	99 ± 7	694 ±56
TYTANIT	124 ± 36	974 ± 49	618 ± 67	1042 ± 183	711 ± 45	286 ± 34	361 ± 54	657 ± 53	58 ± 6	632 ± 91
II term										
Control	230 ± 46	958 ± 21	663 ± 48	1567 ± 103	1076 ± 69	705 ± 96	445 ± 66	867 ± 12	57 ± 4	1052 ± 114
BAP	302 ± 24	1190 ± 206	711 ± 51	1457 ± 155	1009 ± 157	345 ± 55	449 ± 66	1125 ± 183	67 ± 8	1084 ±113
NAA	315 ± 28	1454 ± 254	841 ± 56	1749 ± 159	1150 ± 114	525 ± 87	465 ± 73	1108 ± 106	51 ± 4	1086 ± 183
GA_3	201 ± 21	1324 ± 225	553 ± 40	1283 ± 182	896 ± 75	340 ± 27	409 ± 96	791 ± 29	46 ± 13	1041 ± 230
Cysteina	199 ± 30	1204 ± 145	556 ± 19	1345 ± 168	1008 ± 104	171 ± 35	352 ± 44	829 ± 85	41 ± 6	1006 ± 155
Putrescine	278 ± 60	983 ± 58	538 ± 37	1251 ± 189	794 ± 89	389 ± 83	453 ± 93	706 ± 73	100 ± 19	1081 ± 121
NaCl	229 ± 56	1038 ± 90	411 ± 32	1110 ± 145	717 ± 76	422 ± 21	392 ± 43	567 ± 54	70 ± 17	687 ± 98
ASAHI	158 ± 25	1052 ± 121	526 ± 84	1158 ± 132	806 ± 91	213 ± 69	266 ± 24	671 ± 51	40 ± 5	729 ± 63
TYTANIT	222 ± 88	1423 ± 128	596 ± 57	1247 ± 183	762 ± 38	256 ± 71	298 ± 87	750 ± 30	31 ± 5	781 ± 50

BAP – 6-benzylaminopurine; NAA – 1-naphthaleneacetic acid; cysteine, GA_3 – gibberellic acid, NaCl – sodium chloride), ASAHI SL and TYTANIT – commercial preparates. Data marked with green colour show positive effect of stimulant comparing to control value separative for each control.
