# Supplementary material for: The Effect of Stimulants on Nectar Composition, Flowering, and Seed Yield of Common Buckwheat (Fagopyrum esculentum Moench)
Source: Int J Mol Sci. 2023 Aug 16;24(16):12852. doi: 10.3390/ijms241612852 (PMC10454428; doi:10.3390/ijms241612852)
Supplement: Supplementary file 1 [file ijms-24-12852-s001.zip › Suppl. Table 6 B.pdf]

Supplementary Table S6B. Effect of studied stimulants applied at the beginning of flowering (I term) or at full flowering stage (II term) on content [nM cm^{-3}] following amino-acids: serine, threonine, cysteine, methionine, arginine, aspartic acid, glutamine, glutamic acid, asparagine, lysine in the nectar of common buckwheat line PA16. Nectar was analysed in the open flowers able to fertilization. Means (n = 3) ± SE.

Treatment	Serine	Threonine	Methionine	Asparagine	Aspartic acid	Glutamine	Glutamic acid	Arginine	Lysine
I term									
Control	4192 ± 195	3213 ± 44	95 ± 14	850 ± 10	1712 ± 32	28580 ± 639	2487 ± 110	899 ± 38	1120 ± 48
BAP	1667 ± 99	1157 ± 70	44 ± 2	361 ± 17	705 ± 38	11403 ± 778	1198 ± 68	434 ± 27	472 ± 27
NAA	3508 ± 187	3329 ± 78	58 ± 9	884 ± 80	2273 ± 32	27305 ± 502	3051 ± 19	859 ± 96	733 ± 82
GA_3	3778 ± 115	3196 ± 129	59 ± 7	755 ± 21	1794 ± 41	21345 ± 859	2665 ± 145	908 ± 51	635 ± 25
Cysteine	3143 ± 119	2839 ± 59	35 ± 3	642 ± 25	1695 ± 32	19829 ± 293	2629 ± 130	873 ± 63	1089 ± 59
Putrescine	4666 ± 53	3726 ± 191	97 ± 7	995 ± 11	1819 ± 78	27650 ± 294	2805 ± 104	900 ± 85	1075 ± 50
NaCl	3804 ± 121	1705 ± 34	19 ± 7	701 ± 10	2182 ± 72	19939 ± 467	2893 ± 134	910 ± 39	812 ± 98
ASAHI	3981 ± 83	2645 ± 92	87 ± 6	1022 ± 23	2203 ± 76	30562 ± 500	3000 ± 196	1210 ± 53	1049 ± 86
TYTANIT	3394 ± 161	2620 ± 118	76 ± 5	752 ± 44	1715 ± 63	23385 ± 965	2548 ± 106	785 ± 36	805 ± 52
II term									
Control	2152 ± 99	1374 ± 48	30 ± 11	606 ± 16	1337 ± 48	6653 ± 232	1643 ± 23	281 ± 35	514 ± 64
BAP	2472 ± 43	1605 ± 46	19 ± 5	619 ± 63	1263 ± 74	9246 ± 195	1817 ± 175	275 ± 93	568 ± 20
NAA	3381 ± 154	2059 ± 32	27 ± 2	757 ± 36	2210 ± 68	8370 ± 201	2835 ± 204	177 ± 33	399 ± 56
GA_3	2246 ± 76	1135 ± 80	16 ± 5	410 ± 95	1219 ± 97	5190 ± 107	1875 ± 155	209 ± 41	486 ± 64
Cysteina	2253 ± 65	1100 ± 74	5 ± 1	500 ± 15	1085 ± 37	7281 ± 189	1870 ± 92	187 ± 19	362 ± 64
Putrescine	2176 ± 79	1073 ± 161	29 ± 2	557 ± 25	1193 ± 88	5467 ± 155	1664 ± 126	232 ± 47	429 ± 84
NaCl	1739 ± 96	7510 ± 24	0.9 ± 0.1	313 ± 43	1286 ± 92	5461 ± 152	1895 ± 177	165 ± 12	390 ± 38
ASAHI	2196 ± 99	1365 ± 96	13 ± 8	583 ± 33	1495 ± 67	6423 ± 156	1713 ± 134	180 ± 18	416 ± 67
TYTANIT	1843 ± 91	1237 ± 61	23 ± 3	511 ± 20	1140 ± 51	6894 ± 347	1461 ± 121	133 ± 37	336 ± 60

BAP – 6-benzylaminopurine; NAA – 1-naphthaleneacetic acid; GA_3 – gibberellic acid, NaCl – sodium chloride), ASAHI SL and TYTANIT – commercial preparates. Data marked with green colour show positive effect of stimulant comparing to control value separative for each control.
