# Supplementary material for: The Effect of Stimulants on Nectar Composition, Flowering, and Seed Yield of Common Buckwheat (Fagopyrum esculentum Moench)
Source: Int J Mol Sci. 2023 Aug 16;24(16):12852. doi: 10.3390/ijms241612852 (PMC10454428; doi:10.3390/ijms241612852)
Supplement: Supplementary file 1 [file ijms-24-12852-s001.zip › Suppl. Table 7.pdf]

Supplementary Table S7. Effect of studied stimulants applied at the beginning of flowering (I term) or at full flowering stage (II term) on content [nM cm^{-3}] following polyamines: putrescine, cadaverine, spermidine, spermine in the nectar of common buckwheat lines PA15 and PA16. Nectar was analysed in the open flowers able to fertilization. Means (n = 3) ± SE.

	PA15				PA16			
Treatment	putrescine	cadaverine	spermidine	spermine	putrescine	cadaverine	spermidine	spermine
I term								
Control	36.3 ± 10.6	54.7 ± 0.9	76.6 ± 13.9	27.6 ± 1.9	43.9 ± 5.9	59.3 ± 5.1	87.5 ± 15.4	30.6 ± 1.8
BAP	39.2 ± 2.0	62.2 ± 2.9	93.9 ± 5.4	32.2 ± 1.2	39.7 ± 3.6	59.4 ± 2.1	132.1 ±13.3	43.3 ± 4.7
NAA	47.0 ± 6.4	59.3 ± 3.7	100.1± 29.9	32.7 ± 3.8	34.2 ± 4.5	59.7 ± 0.2	89.8 ± 9.5	35.2 ± 2.2
GA_3	19.2 ± 1.3	53.1 ± 0.4	50.3 ± 4.0	25.3 ± 0.3	36.2 ± 6.6	56.8 ± 2.2	112.6± 14.1	41.8 ± 2.0
Cysteine	35.6 ± 6.4	55.2 ± 0.2	79.5 ± 12.2	28.6 ± 0.7	40.1 ± 0.7	60.6 ± 4.0	113.2 ±14.9	34.3 ± 1.3
Putrescine	28.1 ± 4.3	58.4 ± 4.0	81.3 ± 11.6	31.8 ± 2.4	49.5 ± 4.4	59.9 ± 2.5	93.1 ± 13.3	31.7 ± 2.9
NaCl	33.8 ± 10.1	64.1 ± 0.9	72.7 ± 11.7	29.5 ± 0.7	26.8 ± 4.2	60.2 ± 2.7	90.7 ± 11.8	35.9 ± 4.5
ASAHI	26.0 ± 8.4	55.5 ± 2.8	69.4 ± 8.8	28.7 ± 0.8	68.6 ± 16.6	58.6 ± 3.6	99.7 ± 9.3	34.1 ± 4.4
TYTANIT	37.9 ± 5.8	54.8 ± 0.7	66.9 ± 3.1	27.3 ± 0.9	75.6 ± 17.3	66.2 ± 1.7	117.5 ±5.5	34.5 ± 1.1
II term								
Control	16.6 ± 3.3	59.6 ± 0.4	92.1 ± 2.2	36.7 ± 0.4	17.4 ± 3.2	55.0 ± 0.9	81.4 ± 6.3	32.3 ± 0.7
BAP	16.1 ± 1.8	55.9 ± 0.7	70.5 ± 8.1	27.9 ± 2.9	20.0 ± 7.9	56.3 ± 0.8	61.5 ± 11.3	31.8 ± 2.4
NAA	24.7 ± 4.6	58.1 ± 0.8	103.5 ±10.9	36.6 ± 1.8	15.7 ± 3.8	53.2 ± 0.2	73.4 ± 9.7	32.0 ± 1.2
GA_3	10.9 ± 2.6	54.1 ± 0.1	70.8 ± 6.8	34.6 ± 1.0	18.1 ± 4.5	57.2 ± 1.2	76.3 ± 15.6	36.0 ± 6.6
Cysteina	18.7 ± 4.3	55.2 ± 0.6	85.1 ± 10.5	32.5 ± 3.1	23.8 ± 3.2	58.1 ± 2.5	99.3 ± 8.2	36.1 ± 1.3
Putrescine	23.6 ± 7.2	57.3 ± 2.3	84.4 ± 6.7	34.8 ± 0.6	17.7 ± 2.9	58.7 ± 1.6	78.9 ± 10.3	33.6 ± 3.1
NaCl	17.1 ± 3.7	56.3 ± 0.8	69.7 ± 6.4	29.4 ± 1.7	16.0 ± 4.6	56.2 ± 0.2	72.5 ± 15.6	37.5 ± 5.9
ASAHI	25.3 ± 4.4	54.8 ± 0.8	82.1 ± 13.0	32.4 ± 2.4	18.6 ± 6.0	59.1 ± 0.1	77.4 ± 18.9	32.7 ± 2.7
TYTANIT	10.4 ± 0.3	56.0 ± 0.8	60.0 ± 3.2	28.6 ± 1.8	11.1 ± 0.6	36.3 ± 1.1	60.1 ± 5.1	30.5 ± 1.8

BAP – 6-benzylaminopurine; NAA – 1-naphthaleneacetic acid; cysteine, GA_3 – gibberellic acid, NaCl – sodium chloride), ASAHI SL and TYTANIT – commercial preparates. Data marked with green colour show positive effect of stimulant comparing to control value separative for each control.
